# Gender Differences in Sex Education in China: A Structural Topic Modeling Analysis Based on Online Knowledge Community Zhihu

**DOI:** 10.3390/children9050615

**Published:** 2022-04-26

**Authors:** Wen Shi, Yuxuan Lin, Zihan Zhang, Jing Su

**Affiliations:** 1 School of Journalism and Communication, Jinan University, Guangzhou 510632, China; shiwen@jnu.edu.cn; 2 School of Journalism and Communication, Tsinghua University, Beijing 100084, China; lyx21@mails.tsinghua.edu.cn (Y.L.); zhangzih21@mails.tsinghua.edu.cn (Z.Z.); 3 School of Humanities, Tsinghua University, Beijing 100084, China

**Keywords:** sex education, health communication, online knowledge community, structural topic modeling

## Abstract

Considering the traditional concept of sex in China’s official discourse and lack of social support system for sex education in China, burgeoning Internet knowledge community serves as an important forum for unprepared Chinese young parents to discuss and improve sex education. In this study, we conducted a structural topic modeling analysis of sex education discussions on Zhihu, the biggest online knowledge community in China. We found attention towards sex education are biased in China, where basic sexual terminologies are mentioned, but other important topics such as reproductive health, emotional attachment, and gender identity are insufficient or even absent, failing to fulfill the goal of Comprehensive Sexuality Education (CSE). This study paid special attention to gender differences in discussants, expected educators, and expected receivers of sex education. Findings show that boys are not considered as important sex education objects as girls, although many of them suffered from sexual assault and sexual diseases. They are always mentioned as roles that offend women rather than promoting or protecting themselves through sexual knowledge. Most discussants and expected educators of sex education are women, reflecting men’s lack of attention to sex education issues as both individuals and fathers.

## 1. Introduction

Since the reform and opening up in China, the sexual values and behaviors of Chinese young people have undergone great changes [1]. Numerous reports have brought attention to the public danger posed by the mismatch between Chinese youth’s sexual behavior and their relatively poor sexual knowledge. For example, the low rate of contraceptive use among young people (32.3 percent among unmarried women) has led to a high rate of unmarried pregnancies and induced abortions (28.13 percent). In addition, sexual transmission has become the main route of HIV infection, with 958,000 HIV infections reported in China as of October 2019. In addition, gender-based violence is also widespread, including intimate partner violence (IPV), sexual violence, sexual coercion, and child sexual abuse (CSA), which may be underestimated in China because many victims are afraid to seek help [2].

Although the academic world has reached a consensus that sex education in China is essential, there is still a lack of constructive perspectives on how to carry out sex education in the Chinese context. Sex education in China’s public education system seems unlikely to improve in the short term. As early as 2011, the Program of Action for The Development of Chinese Children (2011–2020) proposed to “integrate sexual and reproductive health education into the compulsory education curriculum system”, but, in reality, it is difficult to systematically carry out or openly discuss sex education in the education system. Due to the traditional conservative social and cultural background of sex in China, Chinese authorities often avoid directly mentioning the term “sex education” [3]. Many schools lack perfect curriculum carriers, and teachers are reluctant to mention the topic of early childhood sex education to parents for fear of embarrassment or suspicion [4].

Considering the traditional concept of sex in China’s official discourse and the uneven educational resources among different regions, the family should be regarded as a more promising place for sex education in China today. A group of notable data shows that, nowadays, more than 90% of Chinese parents are aware of the importance of sex education, but only 62.04% will take the initiative to give sex education to their children [4]. Another set of data worth noting is that Chinese parents who are willing to give sex education to their children do not know more knowledge of sex than those who are not willing to [5]. Considering the positive complementary effect between family sex education environment and school sex education [6], we believe that the current direction of sex education in China is to make young parents who are willing to conduct sex education more knowledgeable and active.

It is not surprising that parents are usually not well-prepared to provide a complete education about sex for their children [7]. However, the worrying fact is that China does not have as complete a social support system for sex education as developed countries [8], so Chinese parents find it difficult to obtain the training and keep up to standards that sex education providers need. To complement this, China’s burgeoning Internet knowledge community has become an important place for young parents to discuss and improve sex education. On Zhihu, China’s largest Internet knowledge community, sex education is a special topic with 93,808 followers and 7529 questions. The discussion here refers to the reflection on the current situation of sex education in China and the comments on social issues related to sexual health and sex crimes. Without sex education entering the official public discourse system, Zhihu’s encouragement of quality discussion and protection of personally identifiable information make it an ideal place for young parents to supplement knowledge, raise awareness, and have communication.

This study aims to characterize Zhihu users’ discussions towards sex education by looking into the distribution of themes. Special attention is paid to gender differences reflected in the discourse of sex education discussions. This is because gender difference is an important component of the Chinese traditional gender concept, which is merged and collided with the increasingly open sexual concept and the rising gender equality consciousness. Therefore, it has repeatedly become a heated topic in public sphere. To be specific, we pay attention to the gender composition of discussants, implementers, and receivers of sex education in the discussions and their preference of sub-topic of sex education. On this basis, we analyze the limitations of individuals’ recognition toward sex education and provide constructive suggestions, in order to promote a more comprehensive, systematic, and healthy sex education in China.

## 2. Literature

### 2.1. Sex Education in the Chinese Social-Cultural Context

According to social constructionism, sexual behaviors with physiological similarities may have different social and subjective meanings [9]. Therefore, although the trend of sexual behavior of Chinese teenagers is increasingly similar to that of western countries, the socio-cultural context of sex education in China still needs to be fully taken into consideration. Traditional Chinese values about sex emphasize childbearing and social stability [10]. Relationships before the age of 18, premarital or extramarital sex that violates Chinese traditional conceptions, gay sex, and masturbation is not acceptable.

Because of cultural background, development stage and many other reasons, sex education as a whole has not been systematically carried out or openly discussed in China. Since “reform and opening up”, among the more than 30 documents concerning sex education have indicated that few Chinese officials directly mention “sex education”, instead, they refer to “puberty education”, “hygiene education”, and “health education” [3].

As early as in 2011, the Program of Action for The Development of Chinese Children (2011–2020) has proposed to “integrate sexual and reproductive health education into the compulsory education curriculum system” [11]. However, the popularity rate, teacher’s attitude, teaching philosophy, and other aspects still need to be improved. A study based on a Shanxi Province found that more than half of kindergartens in Shanxi have not offered any sex education, although the largest number of parents want sex education in schools. In addition, 60.38% of teachers are reluctant to mention the topic related to childhood sex education to parents in parent-school communication occasions due to the possibility of being embarrassed or being questioned [4]. Even in universities, most teachers engaged in sex education do not have professional training, and some of them tend to teach students based on Chinese sex culture and follow traditional concepts [12].

Home has been considered a promising place for sex education, especially in China. Some scholars believe that parents are the most influential people in sex education, and whether parents have a positive attitude towards sex education will affects their children’s enthusiasm in sex education learning at school [5,13]. When parents have correct knowledge of sex education and have good interaction with teachers on children’s sexual behavior, family sex education and school sex education can complement each other [6]. According to a study of the UK, parents are easy role models for their children. A relatively less awkward environment conducive to sex education also needs to be created by parents rather than schools, which may achieve better results [14]. In Chinese families in which mothers are well educated, young people are less likely to engage in risky sexual behavior, such as sexually transmitted infections and accidental pregnancies due to first-time sex without condoms [15]. According to the current survey in China, most Chinese parents believe that parents should be the first teachers of their children’s sex education [16]. In addition, 90% of Shanxi adults believe it is necessary to carry out early childhood sex education, and 62.04% will take the initiative to carry out sex education [4]. However, a study conducted in Shanghai pointed out that, in the early 21st century, the situation of family sex education in Urban China was unsatisfactory [17].

### 2.2. Gender Differences in Family Sex Education

Some studies have found that there are obvious gender differences in the implementation of family sex education, which is mainly reflected in the two dimensions: educator of sex education (father and mother) and receiver of sex education (boys and girls).

Because of some social norms and stereotypes, men who need to support the family are often considered to be not giving full play to fatherhood in family education [18], as well as in sex education [19,20,21]. In general, there may be more sex education between the same gender. Most of the time, mothers talk about sex with their daughters, and fathers talk about sex with their sons [22]. Fathers and daughters talk less [23]. Some cultures believe that it is difficult for fathers to discuss sex with their daughters, which may exacerbate the absence of fathers’ role in family sex education. This may be influenced by religion [24], patriarchy [25], and other factors. Considering the irreplaceable complementary roles of paternity and motherhood in the education of children [26], in family sex education, fathers are required to assume more roles that can only be completed by them. Examples include masculinity [27], how to use condoms [28], etc.

In China, male adolescents are more likely to talk to their fathers, while female adolescents are more likely to talk to their mothers [29]. In a kid’s childhood, mothers participate more in child sex education than fathers [16]). In popular Chinese picture books on children’s sex education, mothers are regarded as storytellers, while fathers are absent visually in picture books, which further legitimizes mothers as playing a major role in taking the responsibility of childbirth and teaching sex knowledge implicitly [30]. In addition, many mothers’ answers of “where do I come from” have difficulty in responding to questions such as “can I be born without father”, which may make children think that the father’s role in the family is not important and thus fail to form a complete understanding of society [31].

According to the Technical Guidelines for International Education, both boys and girls should receive a comprehensive sex education (CSE), which covers the following areas: people’s understanding of the body and the relationship between people themselves and the body, emotional attachment and love, biological sex; gender, social gender identity, sexual orientation, sexual intimacy, sexual pleasure, and reproduction. In reality, however, topics such as sexual health are mainly targeted at girls rather than boys [32]. A Slovak study found that girls were more likely to discuss the responsible attitudes to sexual life, sexual abuse, parenting, contraception and gender equality, while boys were more likely to discuss sex [33]. This difference needs to be remedied by sex education in the later stage. For example, there is a need for girls to be taught about sexual behaviors and for boys to be taught more about responsibility and equality.

In addition, there are differences in the way and purpose of sex education for boys and girls in families. Research conducted in Beijing has shown that boys are less embarrassed than girls in discussing sex with their parents [34]. As girls receive more ascetic education, it is believed that a “no ask, no tell” dynamic is formed between parents and daughters when daughters are just coming of age [35]. Despite this, parents still discuss sex with daughters, and this is usually for girls to protect themselves [36]. Just as Hertzog said: protect the daughter’s reputation about sex, maintain her spousal value, and protect her from sexual assault [35].

### 2.3. Online Community as Sex Education Discussion Forums

Some scholars have pointed out that in most cases, parents are not prepared to provide their children with a complete sex education [7]. In fact, Chinese adults—especially young parents—also need to have “sex education”. Considering that sex education in China has just received official attention in the last 20 to 30 years, current young parents were born in an era when awareness of sex education was weak. A survey aimed at primary school students’ parents in a city of Hubei province showed that although 95% of the respondents were willing to allow their children to receive comprehensive sex education. However, contrary to the expectations of similar western surveys, these parents did not know more about sex education than those parents who did not agree to sex education [5].

In developed countries, social support is an important force for family sex education and health education. The support channels usually include community member training, media publicity, and influencing public policies [37]. This kind of social support is quite insufficient in China, although the content of family sex education has been mentioned in China’s official documents (quote: in May 2019, nine departments including the All-China Women’s Federation and the Ministry of Education jointly issued “National Guidelines for Family Education (Revised)”). However, it does not explain the method and educational context in detail and in depth. Some NGOs have also emerged in China in recent years, but they are mainly concentrated in big cities such as Beijing and Shanghai. Therefore, the actual effect of social support is relatively limited.

China’s rapidly emerging Internet community is expected to offer more help to parents in response to the limitations of social support for sex education in the country. According to some surveys, a significant proportion of the public use “user-generated content” (UGC) social media to seek and share health-related information and exchange social support in Internet communities, including not only Twitter, Facebook and other common social media, but also Yelp, Reddit, and Yahoo, and other knowledge communities [38]. The Internet plays an especially important role in sex-related discussions, with sexual and reproductive health being one of the most important areas of concern for young Chinese. However, due to cultural background, young people are often shy to talk about sex offline, so they hope to query and browse useful information online [39].

Online knowledge communities (OKC) are very popular on the Internet in China in recent years. Such platforms, which combine “knowledge sharing” and “online social”, have indicated the era of mass knowledge production [40]. Different from search engines, online knowledge communities provide users with a personalized platform to express their concerns. Compared with the superficial and emotional remarks on WeChat, Weibo and other social media, the articles on Zhihu provide deeper thoughts and more comprehensive arguments. They usually have high-value density, enabling researchers to have in-depth analysis of knowledge sharing behavior [41].

Studies have confirmed that online knowledge community can not only meet users’ needs for searching relevant information, but also provide users with social support and emotional needs [42]. A study on knowledge sharing in an online health community in China shows that, in addition to general knowledge (such as hospital or doctor information), there is another kind of specific knowledge (such as personal experience) in the Internet community. The latter may be uncomfortable to share but may be particularly valuable for other community members [43].

## 3. Research Questions

This study explores the current discourse of sex education in China based on the Zhihu users’ discussions, with the purpose of understanding their reflection and expectation of sex education.

We pay special attention to the impact of gender differences on the sub-topics of sex education in China. Gender differences are reflected in participants, implementers, and receivers of sex education. We hope to explore which gender has become the main force in constructing the current discourse of sex education, which gender is regarded to be responsible for China’s sex education, and which gender are expected to be more educated in sex education. In this case, the framework deviation of sex education caused by gender differences is observed in particular.

To be specific, this study aims to address the following questions:

RQ1: What are the characteristics of the gender distribution of actors (discussant of sex education, educator of sex education and receiver of sex education) in the discussion of sex education on the Internet?

RQ2: What are the themes of sex education reflected in the discourse of sex education discussion on the Internet?

RQ3: What are the themes of sex education associated with the gender of actors (discussant of sex education, educator of sex education, and receiver of sex education)?

## 4. Data

### 4.1. Data Sources

Launched in January 2011, Zhihu is a Chinese Internet Q&A forum that allows people to share knowledge, experiences, and insights with original content, similar to Quora. “Zhihu” means “know”, and as a comprehensive knowledge sharing community, it has established a community-driven content realization business model, covering topics in science and technology, business, film and television, fashion, culture, and other fields. At present, Zhihu has established diversified media forms including text, video, and live broadcast. 

According to iResearch data, Zhihu users have a good educational background, among which 80.1% have a Bachelor’s degree or above. During the same period, only 20.4% of China’s Internet users have a Bachelor’s degree or above, and TGI (TGI = proportion of Zhihu users with a high degree/proportion of Internet users with a high degree × 100) is as high as 392. According to the analysis, users who receive better education tend to have a broader vision and a stronger need for knowledge and cognition, and, at the same time, the high-quality user group is also the guarantee for Zhihu to produce a large number of high-quality contents (https://report.iresearch.cn/report_pdf.aspx?id=3197, accessed on 13 January 2022).

In terms of operation mechanism, at the micro level, each registered user of Zhihu has a Person Rank (PR), and Zhihu users can discuss about a topic they are interested in. The platform ensures the quality of discussion by authenticating the personal identity of users, properly making them anonymous or real-name, folding answers, etc. At the macro level, Zhihu organizes discussions through a “topic-question-answer” structure, in which topics undertake the function of categorizing discussions. Users can categorize questions under one or more topics by adding tags when or after they raise questions. In the topic interface of the website, the system displays popular discussions, essential discussions, and new questions to be answered. The hot discussion refers to the most popular answer and its question currently, the classic discussion refers to the answer and its question with high recognition during the existence of the topic, and the waiting answer refers to those questions with few answers and still needs to be further discussed by users.

According to statistics, now the topic of “sex education” on Zhihu has 93,808 followers and 7529 questions. On the platform, the topic of sex education is usually defined as education about human reproduction, livelihood, physiological needs, coitus, and other aspects of sexual behavior. The process of creating life is generally described in stages, including conception, embryo and placental development, gestation, and childbirth. In addition, sexually transmitted diseases (STDS) and their prevention and contraception are also covered. The education of sexual physiology and sexual psychology knowledge for teenagers is also an important part, including anatomical knowledge of male and female reproductive organs, physical changes during puberty, the process of procreation, moral education of sex, family planning, eugenic knowledge, etc.

### 4.2. Data Description

To overview “sex education” under the topic of discussion on Zhihu, we use the Python to obtain access to Zhihu API (https://www.zhihu.com/api/v4/), grabbing the “Essential discussion” column under the sex education topic, which is more representative than “hot discussion” and “waiting for the answer”. The data were collected on 18 January 2022, and questions which had been collected in “Essential discussion” before the date were acquired. We got 102 unique questions and went further into the questions screen to capture the answers to these questions. For an overview of the above 102 Essential Questions, we display the top 10 of those questions and their English translation in descending order of the number of answers in Table 1 as examples.

The answers to the above 102 questions include the content of the answers and Supplementary Information (number of likes, number of comments, time for answering). We also pay special attention to the information of the respondents, including their gender, whether they are anonymous, whether they have obtained the certification of Zhihu, etc.

In recent years, Zhihu has introduced a knowledge payment system that allows authors to pay for full answers. In this study, it must be noted that, because few answers were paid and the access to such answers is subject to stricter intellectual property protection, we did not capture them. We finally collected a total of 48,671 answers and then the text of the answer is preprocessed by word cleaning and word segmentation.

## 5. Methods

### 5.1. Classification of Participants in Sex Education

We hope to analyze the characteristics of gender distribution of various actors (discussant of sex education, educator of sex education, and receiver of sex education) in the discussion of sex education on Zhihu. We used the following methods to count and classify the gender of each actor in the answers.

#### 5.1.1. Discussant of Sex Education (Providers of Sex Education Discourse)

When grabbing answers, the server also returns the author information for each answer, including whether the author is anonymous, individual or institution, and gender. In this process, we counted all the non-anonymous accounts and documented their gender. 

#### 5.1.2. Educator of Sex Education (Expected Educators of Sex Education Discourse)

We conducted keyword recognition on the answer text and counted the number of words related to motherhood (N_m), such as ‘my mother’, ‘mother’ and ‘mom’. Also count paternity words (N_f), such as ‘dad’, ‘father’, ‘daddy’. The gender ratio was obtained by calculating r = Log(N_m/N_f). If r > 0, the answer emphasizes motherhood, and if r < 0, the answer emphasizes fatherhood.

#### 5.1.3. Receiver of Sex Education (The Expected Educated in Sex Education Discourse)

We conducted keyword recognition on the answer text and counted the number of words related to the girl (N_g), such as ‘female’, ‘woman’, ‘girl’, ‘female students’ and ‘daughter’. Boy-related words(N_b) are also counted, such as ‘male’, ‘man’, ‘boy,’ ‘male students’ and ‘son’. The gender ratio was obtained by calculating r = Log(N_g/N_b). If r > 0, and it indicates that girls are more frequently addressed in sex education, while r < 0 indicates that the boys are more frequently addressed.

### 5.2. Topic Model of Sex Education Discourse

The topic model is a statistical model of natural language processing developed for uncovering the hidden semantic structures within large corpora. It has been frequently used in web text analysis and has become increasingly popular in social science research [44], and the common topic models include LDA (Latent Dirichlet Allocation) and STM (Structural Topic Model). STM is an unsupervised learning model that was introduced by Roberts et al. [45] as one of the most common probabilistic topic models for computer-assisted text analysis. By comparing STM benefits with LDA, scholars have found that STM outperforms its predecessor model in the clustering performance. More importantly, though the traditional theme model can output the probability that specific words belong to of a particular topic, but the output does not contain confidence interval, so, when the researchers hope to combine the probability distribution of external variables and vocabulary, and then conducting hypothesis testing, the results do not have the statistical confidence coefficient. While the advantage of STM model lies in its covariate that can be integrated in the algorithm model rather than post-mortem analysis, it is thus possible to explore the influence of external factors on the distribution of topics.

In this study, we used the newly created Structural Topic Model (STM) to extract the latent topics in 48,671 answers of the 102 Essential Questions. The appropriate value of k (the fixed number of topics) for a given corpus is user-specified, without an absolute “right” answer [46], which means that the researchers needed to set the value based on the results’ interpretability. After several preliminary tests, we initially started at 3 and eventually specified k = 7. STM calculated the high-probability words of each topic, which were summarized into topics on this basis. Based on this, we conclude the framework of sex education discourse of sex education on the Internet (Q2).

STM allows us to add a theme prevalent covariate into the model to analyze how specific variables affect the distribution of themes. We add the gender of each actor (discussant of sex education, educator of sex education, and receiver of sex education) into the model to explore:

When discussing sex education in China?

What framework do male and female discussants prefer respectively (Q3a)?

What framework are preferred respectively when the educated are boys and girls (Q3b)?

## 6. Results

According to the statistics of actors, there are 19,749 non-anonymous individual users who participate in the discussion of sex education topics, including 10,307 female authors and 9442 male authors; in terms of emphasis on paternal and maternal duties, 7226 authors emphasized maternal duties, 2430 emphasized paternal duties, and 39,015 did not explicitly emphasize paternal or maternal duties. In terms of the attention to girls and boys, 12,071 responses put more emphasis on sex education for girls, 6590 on sex education for boys, and 30,010 showed no gender emphasis.

Table 2 displayed the FREX words in Chinese and English from seven topic clusters produced by STM. FREX words are both frequent and exclusive, so that they are taken as identifying words to distinguish topics. As suggested by the developers of STM [47], we labeled the topic by investigating the words associated with topics and documents associated with topics. Two functions, named “labelTopics” and “findThoughts, plotQuote”, in the stm R package are used to generate high probability words and example documents of each topic.

The main topics include teenage love, sexual enlightenment, sexual health, academic discussions, sex crimes involving minors, sexual-related social norms, and crimes involving children. The theme sexual enlightenment takes the largest proportion, which mainly discusses basic concepts related to sex, such as menstrual flow and concepts about romance. This topic is closely related to the family context, including related words to childhood, family, and hometown.

In addition, *sexual health* and *teenage love* account for about 17% each. The former mainly introduces concepts such as sexual organs and sexual concepts from the perspective of sexual knowledge, and under these themes related diseases and their prevention measures were also mentioned. The latter mainly discussed the love relationship between teenagers, especially in the conservative Chinese school context. This discourse expressed the high praise for good learning in Chinese middle schools and the negative attitude towards love among teenagers. Although teenage love and sexual enlightenment seem to overlap, they show different emphases. The theme of teenage love focuses on the disapproved romance and its hindrance to good learning. In contrast, the theme of sexual enlightenment takes place in the family context and involves more sexual enlightening concepts, such as lingerie and menstrual flow.

In addition, *sex crimes involving minors* and *sexually related social norms* account for about 14% respectively. The former mainly focused on juvenile-related crimes and protection issues, and the age of 14 was emphasized as a special time point for sexual crimes stipulated in Chinese laws. The latter mainly discussed gender issues in social life, such as whether boys can be taken into women’s toilets or bathrooms by their mothers, and appropriate social distancing between cousins of the opposite sex. The topic with the smallest percentage was crimes involving children. In this topic, several news events, investigative reports, and literary works are cited to discuss juvenile protection, some of which are related to sexual crimes.

As for Theme 4, we looked into the original texts, and found many keywords came from academic references as author names or headline words. In other words, it shows the discussants’ efforts of introducing academic evidence into online public discussions. Considering Zhihu’s role as a public forum, citations are quite unusual and serve as scientific resources for sex education discussions. Even if the volume of academic quotes is rather limited, they provide chances for future exploration and conversation. The keywords in Theme 7 show a similar pattern, but they come from news reports rather than academic research. These words indicate respondents’ intention to take minor-related sex crimes into a broader background of minor protection, which seems to be common sense in developed countries, but can be regarded as an enlightened mind in the conservative China context, where many victims do not seek legal remedies for the sake of ignorance and shame.

**Figure 1:**
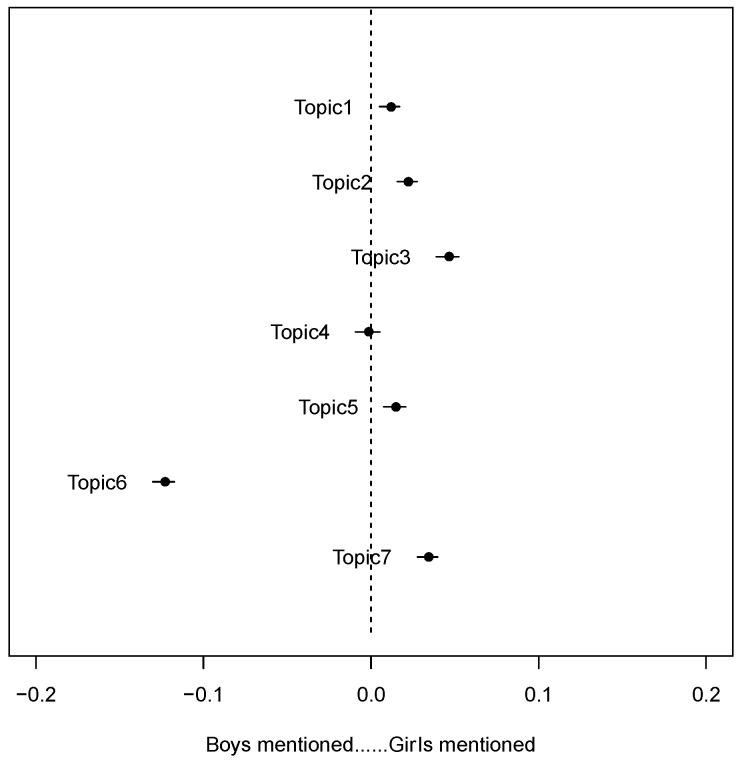
Association between topics and the gender of sex education targets mentioned in the discussions.

We took the variable of whether sex education discussion focuses more on boys or girls as a covariable and analyzed its influence on the topic popularity. The results (Figure 1) showed that in Topic 1 (teenage love), Topic 2 (sexual enlightenment), Topic 3 (sexual health), Topic 5 (sex crimes involving minors), and Topic 7 (crime-related references involving the children), girls were mentioned more times than boys. In Topic 4 (academic discussions), the two are discussed nearly as often. Topic 6 is the only one in which boys are mentioned more often than girls. This topic mainly discusses sexual- related social norms. The phenomenon of a boy brought into the public toilet or bathroom by his mother is a controversial question in the context of China. There is concern that these young boys are not being taught a proper sense of gender boundaries, but, even in this context, boys are referred to as subjects who offend women, rather than subjects who need sex education to improve themselves. 

**Figure 2:**
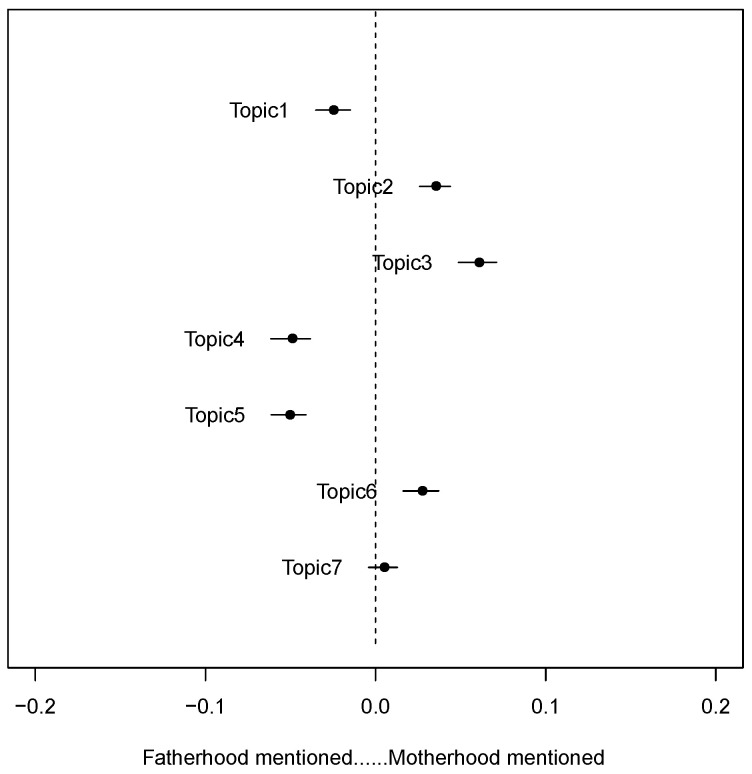
Association between topics and the gender of sex education educators mentioned in the discussions.

We took the variable of whether sex education discussions focus more on paternal and maternal duties as a covariable and analyzed its influence on the topic popularity. The results (Figure 2) showed that, in Topic 2 (sexual enlightenment), Topic 3 (sexual health), and Topic 6 (sexual-related social norms), maternal duty was mentioned more often than fatherhood. This means that mothers are considered to play a crucial role in sex education, whether in terms of providing information about sex to their children or helping them finish the process of gender-related socialization. In Topic 1 (teenage love), Topic 4 (academic discussions), and Topic 5 (sex crimes involving minors), the number of references to the parental duty is significantly higher than that of the maternal duty. Since teenage love is considered inappropriate in the Chinese context, the frequent mention of the parental duty means that fathers are regarded to shoulder more responsibility for disciplining misbehavior.

**Figure 3:**
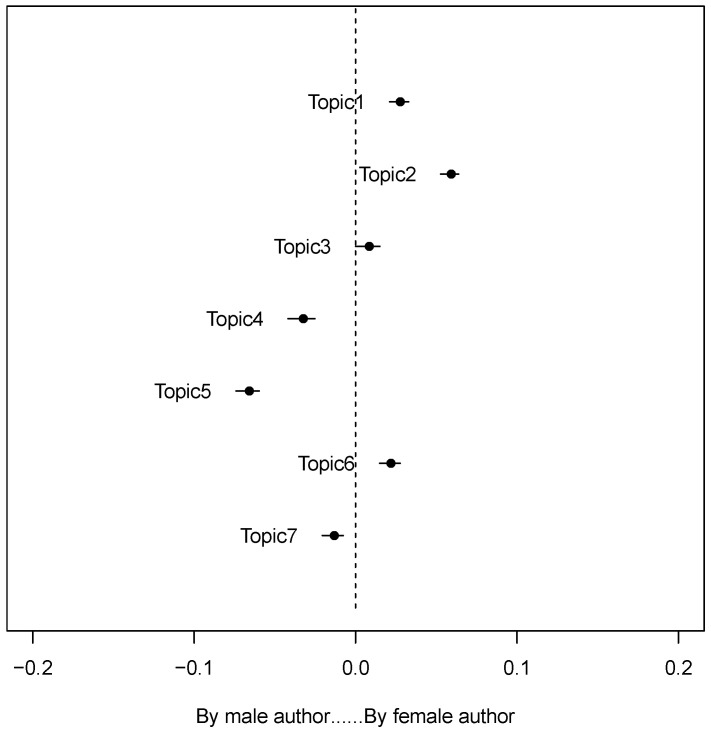
Association between topics and the gender of sex education discussants.

As can be seen from the Figure 3, in discussions on Zhihu, female authors pay more attention to Topic 1 (teenage love), Topic 2 (sexual enlightenment), Topic 3 (sexual health), and Topic 6 (sexual-related social norms), while male authors focused more on Topic 4 (academic discussions), Topic 5 (sex crimes involving minors), and Topic 7 (crime-related references involving the children). Therefore, it can be inferred that male authors are more concerned with academic discussions and social homicide cases, but less interested in how children are educated in everyday settings.

## 7. Discussion

Framework refers to the internal structure hidden in the text and maps the ideas by which these materials are organized. Based on these structures and ideas, the text can select, emphasize, and exclude certain aspects of information. In the social sciences, the essence of framing is considered to be a selective reconstruction of reality by individuals and organizations, as well as a selective expression or emphasis of facts, which often means giving a problem a different definition, solution, or causal relationship [48]. Based on the frame theory, we assume that a topic framework of sex education discussion on Zhihu can reflect users’ attention to some aspects of sex education and neglect of others. In addition, a framework also reflects users’ preferences on attribution and solutions to current sex education problems. By examining the frameworks of sex education, we can describe Zhihu users’ reflections and an ideal blueprint of sex education.

### 7.1. Chinese Sex Education Sub-Topics and CSE

The sub-topic of sex education is biased and has deficiencies, which does not meet the requirements of Comprehensive Sexuality Education (CSE) mentioned in the UNESCO Sustainable Development Goals. According to International Technical Guidance on Sexuality Education (ITGSE), CSE’s requirements are as follows: (a) the understanding of, and relationship to, the human body; (b) emotional attachment and love; (c) sex; (d) gender; (e) gender identity; (f) sexual orientation; (g) sexual intimacy; and (h) pleasure and reproduction. Sex education in China is not evenly distributed in the above-mentioned aspects.

If we re-examine the seven topics discussed above in light of this ITGSE framework, we will find that the eight key concepts of this framework have many congruences with the STM results. For example, “teenage love” (Topic 1) is relevant with “(b) emotional attachment and love”; “sexual-related social norms” (Topic 6) focuses on “(d) gender” and “(e) gender identity”; and “sexual enlightenment” (Topic 2) can be related to all the eight concepts. Table 3 displayed correspondence between the ITGSE framework and themes (or, topics) produced by the STM model. 

It appeared that “sexual enlightenment” and “sexual health”, the two topics which we drew from Zhihu data, matched the most with ITGSE framework. This to some extent can tell that young parent Zhihu users had already focused and not done bad in these two parts of Comprehensive Sexuality Education (CSE). In addition, according to our data analysis, in Topics 2 and 3, more girls and motherhood part were mentioned by female authors.

When we tried to find out about the related parts with boys, fatherhood, and male authors, it came out that the most corresponding key concept of ITGSE framework is “sex”. It is the same with the literature review above where former research discovered that boys are more directly taught about sex itself while girls are more often the audience of a more comprehensive sex education, particularly on how to protect themselves.

Although the STM analysis based on Zhihu data cannot be specified to the very eight concepts of ITGSE framework, the two structures are more or less corresponding with each other. Moreover, with this framework, we could see the gender difference of sex education conducted in today’s China—exactly what we mainly talked about in this article. It is possible to say that similar results could make our discovery more credible.

Compared with the expectations for sex education in ITGSE, some expectations are missing in the seven topic clusters in this study. Taking relationship management as an example, relationships may affect mental health, but the current discussion is still limited to reproductive health. In addition, a homo-sexual relationship is not stable in the clustering results when there is not enough information on sexual minorities and their health risks. It reflects a relatively conservative social environment in China that confirms the claim that traditional sexual values do not easily attach importance to content unrelated to fertility and social stability [10]. Issues related to sexual health and sexual crime have received some attention, but there is still inadequacy in both. As a concern for many Chinese young people [39], sexual health has not yet formed a strong discussion atmosphere on Zhihu. For example, issues such as HPV have not been fully discussed (only 29 out of 48,671 discussions mentioned HPV) or reflected in the clustering results. In discussions related to sexual crimes, most of the keywords are about basic facts of the case while almost none for prevention, response, and assistance. The physical or mental injury caused by the case is rarely mentioned. The loss management is not adopted from the perspective of public health. With the progress of the times, it is necessary to gradually change the values of sex education providers around these missing aspects.

### 7.2. Gender Differences in Chinese Sex Education

After presenting the Top10 of the Essential Questions in descending (in Table 1), we should realize that the list itself also reflects some cognitive tendencies and characteristics of sex education in China. The number one question is “how deficient is sex education in China? what impact might this have?”, receiving 8321 responses. Through the observation and analysis of the answers, it is found that the questions tend to be female-oriented. That is, most discussions are centered on the answers to girls’ life experiences, which include girls getting pregnant before marriage, misbehaving, and misunderstanding sexual life, becoming victims of sexual assaults due to a lack of sex education. Under this tendency, men are often portrayed in discussions as perpetrators and cheaters, and they are also seen as less affected by the lack of sex education. In addition to differences in receivers of sex, educators also differ in the degree to which they discuss. Since the question itself has a negative orientation, many discussants accuse their parents of being conservative, ignorant, and unenlightened in their answers. However, mothers were obviously mentioned more often. Their characters are mostly ones who do secondary harm to their children, such as berating their daughters for being unchaste, accusing their daughters of having abortions or refusing to teach them knowledge about sex and being ashamed of it. We can see from this answer the biased themes and imbalance in terms of gender roles.

Basic gender norms are still the controversial focus under the topic of sex education. With the exception of some questions from a holistic perspective, most of the questions are related to women, and the only one that had a male orientation is the fifth “how do you view the behavior of some mothers bringing their little boys into the women’s locker room to change clothes?”. From an educator’s point of view, the critique of mothers and fathers is balanced on this issue. Many of the discussants blamed the mother for poor parenting, but, at the same time, many discussants were acutely aware of the deeper problem of a lack of paternal responsibility. That is, because fathers have less time to take care of their children, mothers have to take their boys into the ladies’ room because there is no one else to look after them. Many mentioned “widowed parenting” in their answers, arguing that fathers’ educational responsibilities must also be addressed.

STM analysis also confirms that boys are not considered as important sex education objects as girls. In most topics, boys are mentioned less than girls, which is consistent with the empirical research twenty years ago in Shanghai [17]. This also reflects a premise of Chinese sex education, that is, girls are the loser in sexual relationships, and inappropriate sexual behaviors can easily make girls lose what Hertzog calls “spousal value” [35]. Therefore sex education aims to teach girls to protect themselves [36]. However, boys are considered more likely to take advantage. They also seem more comfortable when receiving sex education and discussing sexual behavior [34].

Even in the topics like social norms, boys are mentioned as roles that offend women rather than promoting or protecting themselves through sexual knowledge. Boys do not receive enough targeted knowledge for their gender. In fact, boys also face sexual assault and sexual diseases. However, such risks are yet less-represented in today’s China. Even in a heterosexual relationship, males should be responsible for healthy relationships and the well-being of their partners. However, in current sex education, boys are exempted from this responsibility, as foreign studies have pointed out, sexual health and responsible sex behavior is even more of a target for girls [33]. In particular, the risks of Men Who Have Sex with Men (MSM) behavior are still less of a concern and have not received the attention it deserves. At present, the correlation between the HIV infection rate of MSM in China and factors such as age, education level, and unprotected sex suffer a lack of powerful reports [49].

STM analysis also reveals that, in Internet knowledge communities like Zhihu, most participants and discussants of sex education are women. It means the group that is currently paying attention to sex education and communicating through the Internet is mainly women. The reasons are worth reflecting on and discussing for improvement.

From a macro perspective, gender differences may be a factor hindering the further development of Chinese sex education. In a society with benign interaction, the gender gap in discussion reflects social imbalance of topic attention. In some cases, this difference comes from men and women’s preference of the topic. As for sex education, which should be a field with equal coverage of both men and women, an imbalance reflects the lack of attention by men to gender issues. The reason may be similar to that of the missing fatherhood in family education. Like the lack of jobs, it is related to the stereotyped social division of labor that considers male as breadwinners [18]. This study found that, among the topic categories of sex education topics displayed in the Internet, the topics related to girls are much higher than those of boys. However, previous research has pointed out that sex education between the same-gender family member is more than that of cross-gender [22,29]. Thus, it can be inferred that girl-related topics are more likely to engage women as same-gender sex education providers. In addition, because women tend to receive more sex education that emphasizes danger and responsibility [32], topics such as sexual abuse, parenting, contraception, and gender equality are also more engaging for female discussants.

From a meso-level perspective, in a family with positive interactions, both parents should have basic knowledge on sex education and play a positive and complementary role in providing sex education to their children [26]. Our research also confirms this tendency. Mothers should take on the role of sex education, whether it is to provide their children with sexual knowledge or to help their children properly socialize in relation to their gender, while fathers should take more responsibility for disciplining their children for sexual behavior.

From a micro perspective, group differences in virtual community discussions are also a collection of individual cognitive differences. Women are more active, either as adult women reflecting on the lack of sex education themselves, or as mothers or potential mothers for their children’s sex education, while men’s low individual participation reflects their lack of reflection. The deficiency also shows the lack of male willingness to participate in sex education, consistent with the absence of fatherhood in children’s sex education [16,30].

### 7.3. Sub-Topic Competition in Chinese Sex Education

In addition, the competing discourses between topics under the topic of sex education give us some thought about discussion in the online knowledge community. From the “Top10 of the Essential Questions in descending order of the number of answers (Table 1)” and “Themes and FREX Words produced by STM Model (Table 2)”, we can find that most problems under the topic of sex education are caused by social events, so sex crimes and violations of social norms occupy a large proportion of the discussion, such as Q3 (it is suspected that a 10-year-old girl in Dalian was killed by a 13-year old male student investigation going?) and Q4 (my son Molested a female classmate at school, should I take the initiative to apologize?) in Table 1, and related themes in Table 2 (sex crimes involving minors, sexual-related social norms, and crime-related references involving children). Therefore, the discussion tends to be more extreme problems and intensified contradictions, which will squeeze the discussion space of some daily education issues. Everyday education topics tend not to attract wide attention and discussion in the field of sex. In addition, the sexual part of the relationship is also repeatedly mentioned. People will pay attention to love-related problems like “how do you view the phenomenon of ‘puppy love’?”, and Topic 1 (teenage love) and Topic 2 (sexual enlightenment) take the largest proportion. It dilutes the discussion of education to some extent, but, at the same time, it reflects the way in which people deal with gender issues with a lot of questions and confusion, and then choose to ask questions on social media, which is also one of the manifestations of people’s lack of sex education in the Chinese context.

Due to the limited carrying capacity of the public agenda, agenda-setting is a zero-sum game [50], in which issues compete for media and public attention. In light of this idea, among the competing discourses, there must be some that are weakened, even though they are too important to be neglected. In our cases that are mentioned above, everyday education topics are such weakened discourse in the zero-sum competition.

As an online knowledge community serves as an important forum for young Chinese parents to discuss and improve sex education, the bias in topics and gender roles in discussions may raise the uncertainty of future sex education in China, by reflecting and reinforcing misleading opinions. Although it can be assumed that adults willing to participate in online sex education discussion have higher awareness of its importance than the average, current discussions may fail to equip them with proper knowledge about sex education, allowing for the risk of mis-concepts passed on from generation to generation in family.

## 8. Conclusions

This study uses an automation approach to extract the topic framework of Chinese online sex education discussions on Zhihu. We explored characteristics of the gender distribution of actors (discussant of sex education, educator of sex education, and receiver of sex education) in sex education. Overall distributions of themes, as well as different actors’ associations with specific themes, are quantitively examined.

The findings confirmed that current discussions of sex education in China are biased. Basic sexual terminologies are mentioned, while broader topics such as gender identity and emotional attachment are absent. Even for topics which have drawn some attention, such as sexual health and sexual crime, there is still inadequacy in comprehensive understanding. Gender difference of sex education discussants, educators, and receivers is an important factor influencing the framework of the discussions. Girls are more likely to be targeted as sex education receivers, while the importance of education for boys is under-estimated, even when they are also exposed to sexual assault and sexual diseases in the current situation. Overwhelming female discussants and educators indicates Chinese men’s lack of engagement in sex education issues, both as individuals and fathers. We suggest that dealing with biased themes and imbalance in terms of gender roles is an essential driving force for China’s sex education improvement.

## 9. Limitations

Compared with other well-known platforms such as Weibo and WeChat, Zhihu is prominent for its well-educated discussants and high-quality contents. Its topic–question–answer structure allows for a large volume of sex education discussions taking place in an organized way, making Zhihu a most valuable window to look into the current situation of Chinese sex education. However, the properties above may bring some limitations for our study at the same time. Discussions above on Zhihu can better represent young parents who have a higher educational background and are more concerned about the issue, rather than representing parents in a more demographical way. It means that our discovery is relatively cutting-edge; however, the real situation of family sex education in China may be more serious than discussed in this article. In addition, analyzing Zhihu data can only focus on what people discuss, but it is difficult to focus on what and why people ignore, and their views on topics they are not aware of. In particular, the current STM analysis has difficulty carrying out cross-language analysis, which makes it impossible for us to compare discussions across similar platforms in other countries with Zhihu data. It limits the possibility for us to conduct more in-depth discussions on Zhihu data through comparison. 

## Figures and Tables

**Table 1:** Top 10 essential questions under the topic of “Sex Education” on Zhihu (in descending order of number of answers).

Rank	Question Titles in Chinese	Question Titles in English	Number of Answers
1	中国的性教育有多匮乏?这可能造成哪些影响?	How deficient is sex education in China? What impact might this have?	8321
2	为什么现在有一些初高中的女孩子越来越不爱惜自己的身体?	Why are some girls in junior high and senior high school less and less caring about their bodies?	7506
3	大连10岁女孩疑被13岁男学生杀害一事, 案件调查进展如何?	It is suspected that a 10-year-old girl in Dalian was killed by a 13-year-old male student. How is the investigation going?	3725
4	表兄妹之间应不应该避嫌?	Should male and female cousins behave to avoid suspicion?	2936
5	如何看待部分母亲带小男孩进女更衣室换衣服的行为?	How do you view the behavior of some mothers bringing their little boys into the women’s locker room to change clothes?	1873
6	儿子在学校猥亵了女同学, 我该主动去道歉吗?	My son molested a female classmate at school, should I take the initiative to apologize?	1840
7	如何评价新闻「一位妈妈声称学校发的《小学生性健康教育读本》尺度太大」?	How to comment on the news “A mother claims that the Sexual Health Education Reader for Primary School Students issued by the school is too explicit”?	1461
8	在上高中的女儿卧室里无意看到一套情趣内衣, 内心很气愤, 要怎么与她沟通, 要不要告诉妻子?	I accidentally saw a set of erotic underwear in my daughter’s bedroom, who is still in high school, and I was very angry. How should I communicate with her, or should I tell my wife?	1428
9	如何看待「早恋成风」的现象?	How do you view the phenomenon of “puppy love”?	1326
10	如果自己的孩子是同性恋, 该怎么办?	What if my child is gay?	1030

**Table 2:** Themes and FREX words produced by the STM model.

Num.	Themes	FREX Words	FREX Words in English	Proportion
1	teenage love	谈恋爱, 初高中, 男同学, 好好学习, 小混混, 晚自习, 重点高中, 高中同学, 努力学习, 前女友	Dating, junior high school, male classmate, study hard, gangster, evening self-study, municipal high school, high school classmate, study hard, ex-girlfriend	0.175
2	sexual enlightenment	女孩子, 男朋友, 小时候, 小姑娘, 情趣内衣, 大姨妈, 家里人, 四年级, 前男友, 回老家	girl, boyfriend, childhood, little girl, lingerie, menstrual flow, family, fourth grade, ex-boyfriend, back home	0.203
3	sexual health	性教育, 性行为, 性知识, 避孕套, 艾滋病, 进行性, 性器官, 安全套, 生物课, 性观念	sex education, sexual behavior, sexual knowledge, condoms, AIDS, Conduct sex-related behaviors, sexual organs, condoms, biology class, sex concept	0.176
4	academic	提问者, 研讨会, 医科大学, 妊娠期, 毒理学, 海峡两岸, 李园园, 汪晖期, 行业标准, 广西大学	Questioner, Seminar, Medical University, Pregnancy, Toxicology, Cross-Strait, Li Yuanyuan, Wang Huiqi, Industry Standards, Guangxi University	0.102
5	sex crimes involving minors	未成年人, 未成年, 受害者, 保护法, 刑事责任, 受害人, 监护人, 十四岁, 被害人, 杀人犯	minor, underage, victim, protection law, criminal responsibility, victim, guardian, fourteen, murderer	0.141
6	sexual-related social norms	更衣室, 小男孩, 表兄妹, 卫生间, 女厕所, 游泳馆, 女浴室, 女孩儿, 男厕所, 洗手间	locker room, little boy, male and female cousins, toilet, women’s toilet, swimming pool, women’s bathroom, girls, men’s toilet, restroom	0.143
7	Crime-related References involving children	调查报告, 詹姆斯, 谋杀案, 巴杰尔, 刘文利, 芦鸣祺, 杨素萍, 前列腺, 房思琪, 林奕含	Investigation Report, James, Murder, Bulger, Liu Wenli, Lu Mingqi, Yang Suping, Prostate, Fang Siqi, Lin Yihan	0.061

**Table 3:** Correspondence between ITGSE framework and themes produced by the STM model.

Num.	Themes	ITGSE Key Concept
Topic 1	teenage love	(b) emotional attachment and love.
Topic 2	sexual enlightenment	All eight concepts.
Topic 3	sexual health	(a) the understanding of, and relationship to, the human body; (c) sex; (f) sexual orientation; (g) sexual intimacy; (h) pleasure and reproduction.
Topic 4	academic	N/A
Topic 5	sex crimes involving minors	(c) sex.
Topic 6	sexual-related social norms	(d) gender; e) gender identity.
Topic 7	Crime-related References involving children	(c) sex.

## Data Availability

The data presented in this study are available in Supplementary Material here.

## Supplementary Materials

The following supporting information can be downloaded at: https://www.mdpi.com/article/10.3390/children9050615/s1, Supplementary material: study data.
